# CVVHD results in longer filter life than pre-filter CVVH: Results of a quasi-randomized clinical trial

**DOI:** 10.1371/journal.pone.0278550

**Published:** 2023-01-11

**Authors:** Lewis Mann, Patrick Ten Eyck, Chaorong Wu, Maria Story, Sree Jenigiri, Jayesh Patel, Iiro Honkanen, Kandi O’Connor, Janis Tener, Meenakshi Sambharia, Mony Fraer, Lama Nourredine, Douglas Somers, Jonathan Nizar, Lisa Antes, Sarat Kuppachi, Melissa Swee, Elizabeth Kuo, Chou-Long Huang, Diana I. Jalal, Benjamin R. Griffin

**Affiliations:** 1 Division of Nephrology & Hypertension, University of Iowa Hospitals & Clinics, Iowa City, Iowa, United States of America; 2 University of Iowa Institute for Clinical and Translational Science, Iowa City, Iowa, United States of America; 3 Center for Access & Delivery Research & Evaluation (CADRE), Iowa City Veterans Affairs Health Care System, Iowa City, Iowa, United States of America; University of Texas Medical Branch at Galveston, UNITED STATES

## Abstract

**Background:**

Filter clotting is a major issue in continuous kidney replacement therapy (CKRT) that interrupts treatment, reduces delivered effluent dose, and increases cost of care. While a number of variables are involved in filter life, treatment modality is an understudied factor. We hypothesized that filters in pre-filter continuous venovenous hemofiltration (CVVH) would have shorter lifespans than in continuous venovenous hemodialysis (CVVHD).

**Methods:**

This was a single center, pragmatic, unblinded, quasi-randomized cluster trial conducted in critically ill adult patients with severe acute kidney injury (AKI) at the University of Iowa Hospitals and Clinics (UIHC) between March 2020 and December 2020. Patients were quasi-randomized by time block to receive pre-filter CVVH (convection) or CVVHD (diffusion). The primary outcome was filter life, and secondary outcomes were number of filters used, number of filters reaching 72 hours, and in-hospital mortality.

**Results:**

In the intention-to-treat analysis, filter life in pre-filter CVVH was 79% of that observed in CVVHD (mean ratio 0.79, 95% CI 0.65–0.97, p = 0.02). Median filter life (with interquartile range) in pre-filter CVVH was 21.8 (11.4–45.3) and was 26.6 (13.0–63.5) for CVVHD. In addition, 11.8% of filters in pre-filter CVVH were active for >72 hours, versus 21.2% in the CVVHD group. Finally, filter clotting accounted for the loss of 26.7% of filters in the CVVH group compared to 17.5% in the CVVHD group. There were no differences in overall numbers of filters used or mortality between groups.

**Conclusions:**

Among critically patients with severe AKI requiring CKRT, use of pre-filter CVVH resulted in significantly shorter filter life compared to CVVHD.

**Trial registration:**

ClinicalTrials.gov, NCT04762524. Registered 02/21/21—Retroactively registered, https://clinicaltrials.gov/ct2/show/NCT04762524?cond=The+Impact+of+CRRT+Modality+on+Filter+Life&draw=2&rank=1.

## Introduction

Continuous kidney replacement therapy (CKRT) is used in critically ill patients with severe acute kidney injury (AKI). Because it is associated with less hemodynamic instability than intermittent hemodialysis, CKRT is the most commonly utilized form of kidney replacement therapy in the intensive care unit (ICU) setting [1]. Filter loss due to clotting impacts about a quarter of all filters used during CKRT [2] and results in a decrease in time on treatment, reductions in daily delivered effluent dose, and blood loss if the filter is not rinsed back in time [3]. As a result, filter life has been identified as a key CKRT quality metric [4, 5].

Multiple factors are involved in filter life, including access location, anticoagulation use, and factors related to the patient’s illness [6], but an additional and understudied component is CKRT modality. CKRT can be delivered using convective clearance, as in continuous venovenous hemofiltration (CVVH) or diffusive clearance as in continuous venovenous hemodialysis (CVVHD). CVVH can be delivered using pre-filter or post-filter replacement fluid, or a combination. Use of post-filter replacement fluid increases the circuit’s filtration fraction (FF), which measures the degree of hemoconcentration through a CKRT filter. Higher rates of ultrafiltration and fluid removal across the filter result in more concentrated blood at the end of the filter, theoretically predisposing to increased clotting [7]. Whether filtration fraction also applies in pre-filter CVVH is a matter of debate [8].

However, while hemoconcentration may not occur to a high degree in pre-filter CVVH, there are still rapid fluid shifts that result in sizeable concentration fluctuations in hematocrit and plasma protein levels over a short period of time within the CKRT circuit, which may predispose to increased clotting. The use of dialysate (diffusive clearance) as opposed to post-filter or pre-filter replacement fluid results in substantially less hemoconcentration and substantially smaller shifts in hematocrit and plasma protein concentrations, respectively. Indeed, existing evidence suggests that filter life may be longer when using diffusive clearance, although direct comparisons to pre-filter CVVH are lacking [9–12].

The purpose of this study was to examine filter life in patients treated with either pre-filter CVVH or CVVHD, with the hypothesis that filter life would be longer in patients treated with CVVHD due to the lack of either hemoconcentration or rapid concentration shifting across the filter.

## Materials and methods

### Trial design

This study was a single center, pragmatic, unblinded, quasi-randomized cluster trial in which the use of CVVHD was compared with pre-filter CVVH among critically ill adults with severe AKI at the University of Iowa Hospitals and Clinics (UIHC) between March 2020 and December 2020. The study was considered quasi-randomized because time-based clusters alternated between modalities.

### Participants

All adults 18 year of age or older with severe AKI requiring CKRT during the trial period were enrolled at the time of CKRT initiation. This study included patients within all ICUs at UIHC, which included the medical, cardiac, and surgical/neurosurgical ICUs. Patients were excluded if they were on extracorporeal membrane oxygenation (ECMO) at the time of CKRT initiation.

### Interventions

Patients were assigned based on date of CKRT admission to receive either pre-filter CVVH or CVVHD as their initial CKRT treatment modality. In order to maximize patient safety during critical illness, the nephrology consulting team was allowed wide latitude in the prescription of effluent flow rates, fluid composition, and anticoagulation. However, standard practice at the UIHC, based on KDIGO guidelines [13], is to prescribe an effluent flow rate of 20–25 mL/kg/hr with regional citrate anticoagulation unless the patient is on a systemic heparin infusion, using one of four pre-packaged fluid compositions. We use a 3% ACD-A Citrate formulation, which has a citrate concentration of 112.9 mmol/L. Standard practice at our institution is to start at 150 mL/hour, and titrate to achieve a post-filter calcium of <1.6 mg/dL. All patients were prescribed a post-filter replacement fluid flow rate of 200 mL/hour, as recommended in Prismaflex machines to minimize the blood-air interface in the bubble trap. Prismaflex filters with AN 69 dialyzer membrane (Acrylonitrile and sodium methallyl sulfonate copolymer) were used. Filters are typically changed every 72 hours at UIHC, but were allowed to run for up to 120 hours to preserve supplies during the COVID-19 pandemic. Fellows writing CKRT prescriptions were educated on the dilutional impact of pre-filter CVVH on dosing. After initiation, all adjustments to the prescription, including a change of modality if deemed necessary, were at the discretion of the nephrology consult service. Enrolled patients who were discharged and then readmitted, or were off CKRT for more than a week and then restarted, were restarted on the modality indicated by the block at the time of re-initiation. For this reason, a small number of patients (n = 4) appear in both groups. Data was collected electronically upon discontinuation of CKRT. The primary outcome was filter life, and secondary outcomes were number of filters used, number of clotted filters, number of filters reaching maximum lifespan, days of therapy, and in-hospital mortality.

### Outcomes

The primary outcome was the adjusted mean ratio of mean filter life in pre-filter CVVH compared to CVVHD. Secondary outcomes were number of filters used, number of CKRT days, number of filters lasting >72 hours, and in-hospital mortality.

### Sample size

Based on previous quality data from 2019, we anticipated a filter life of 32 hours with a standard deviation of 25. Using a 2-sided α value of 0.05 and a power of 80% to detect a 20% difference between groups, 546 filters were required. We anticipated 4 filters per patient on average giving a sample size of 137 patients.

### Randomization

This pragmatic trial was organized into 20 alternating 2-week blocks, which corresponded to the 2-week blocks in which UIHC renal fellows serve on the ICU rotation. This design was chosen to lessen delays or confusion related to decisions of modality initiation in this procedure, which is usually initiated on an emergency basis in critically-ill patients. Pre-filter CVVH was randomly selected by coin toss for block 1. For each two-week block of the trial, the nephrology consulting team would start the patient on either pre-filter CVVH or CVVHD according to the date of CKRT initiation. Once patients were assigned to a modality, they remained on that modality until their CKRT treatment was ended or until the Nephrology consult team felt a modality change was necessary based on clinical judgment. Due to an accidental block repeat of pre-filter CVVH (blocks 9 and 10), the trial ultimately ended with 11 blocks of pre-filter CVVH and 9 blocks of CVVHD. This trial was approved by the institutional review board at UIHC with a waiver of informed consent. The trial was registered at clinical trials.gov (NCT04762524), and this paper was constructed using the CONSORT guidelines for clinical trials.

### Blinding

Due to the nature of the procedure, which requires careful and frequent adjustments to machine parameters based on a patient’s clinical course, provider blinding was not feasible.

### Statistical methods

Analyses were conducted at the level of the individual filter, in both an intention-to-treat and per-protocol fashion. Continuous variables are reported as means and standard deviations or as medians and interquartile ranges; categorical variables are reported as frequencies and proportions. The primary analysis compared the average filter life in the pre-filter CVVH and CVVHD groups in an intention-to-treat fashion. Because the distribution of filter life was not normal, and filters are nested within patients, generalized linear mixed models (GLMM) with gamma distribution was used with a log link function and a random intercept. Because of the wide latitude given to providers, there were limited instances where the modality was changed during the course of CKRT. Primarily, this resulted in a change to CVVHDF with a combination of pre-filter and dialysate fluid. A secondary analysis compared filters in the pre-filter CVVH and CVVHD groups based on actual modality delivered after removal of CVVHDF filters. A filter survival curve was generated for filter time in the primary analysis. All statistical tests were considered to be significant at a 2-sided p < 0.05. All analyses were performed using SAS software version 9.4 (SAS Inc., Cary, NC).

### COVID-19 analysis

An additional unplanned analysis, which was added shortly after trial initiation in the setting of the COVID-19 pandemic, was to evaluate COVID-19 filters vs. non-COVID-19 filters. Given the differential anticoagulation strategies used in response to COVID-19, we also added additional unplanned analyses for anticoagulation strategy. No patients in this trial developed COVID-19 after CKRT initiation, and so all patient filters in patients who had tested positive for COVID-19 were considered COVID-positive filters. Continuous variables are reported as means and standard deviations or as medians and interquartile ranges; categorical variables are reported as frequencies and proportions. Mann-Whitney U test and chi-square test were used accordingly. Due to the significant impacts of anticoagulation and COVID-19 on filter life, additional analyses were conducted to determine if significant statistical interactions were present. We followed a backwards selection procedure to remove higher-order interactions that were not significant, resulting in a model that only had modality in it. For both the ITT and PP predictors, we fit univariate models, then added group and the modality*group interaction. Anticoagulation was stratified into heparin-only and citrate-only.

## Results

### Baseline characteristics

**Fig 1:**
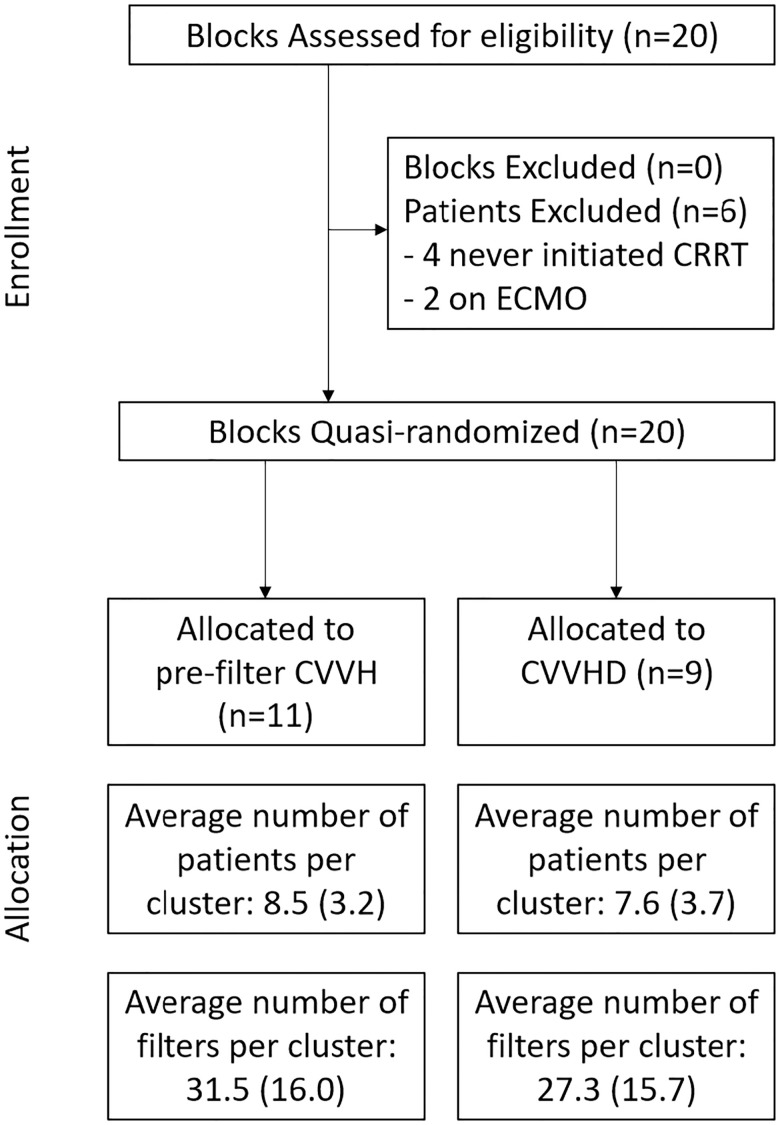
Participant flow diagram.

In all, there were 591 filters used over 161 patient-sessions (157 unique patients) in the trial (Fig 1). Table 1 describes the baseline characteristics of the patients based on initial assigned treatment modality, and also gives reasons for filter loss. There were 346 filters and 94 patient-sessions in the pre-filter CVVH group compared to 245 filters and 67 patient-sessions in the CVVHD group. In the per-protocol analysis, there were 293 and 230 filters in the pre-filter CVVH and CVVHD groups, respectively. The differences in group size were the result of having 11 pre-filter CVVH blocks compared to 9 CVVHD blocks. The most common reason for the differences between ITT and per protocol analyses were changes from pre-filter CVVH or CVVHD to CVVHDF. A total of 47 filters (13.6%) in the CVVH group were switched to CVVHDF at the nephrology team’s discretion, and was frequently driven by higher filter pressures in the pre-filter CVVH group. Changing from CVVHD to CVVHDF was seen in only 17 filters (6.9%). Four patients (2.5%) who used a total of 26 (4.4%) filters were included in both categories based on having two distinct CKRT treatments that fell in different blocks. There were no differences between the groups in terms of age, gender, race, or underlying comorbidities, and the sequential organ failure assessment (SOFA) score was equivalent. Use of anticoagulation was similar between groups, and in each group approximately 10% of patients were treated without anticoagulation. COVID-19 infections, an unanticipated factor when the trial was designed, impacted nearly a third of all AKI-CKRT patients, but the rate of infection appeared to be balanced between the two groups. These patients were generally treated with dual-anticoagulation (defined as regional citrate anticoagulation with systemic heparin), with no significant difference in COVID anticoagulation strategy by group.

**Table 1:** Baseline characteristics of intention-to-treat analysis of patients by modality.

*Characteristics* *	*CVVH (n = 94)*	*CVVHD (n = 67)*	*P value*
	*N (%)*	*N (%)*	
Female	29 (30.9)	28 (41.8)	.15
Age, years, mean (SD)	57.4 (15.4)	57.3 (15.8)	.96
Caucasian	73 (77.7)	47 (70.1)	.28
Body mass index, mean (SD)	33.3 (9.0)	33.0 (9.7)	.87
Comorbidities			
Chronic Kidney Disease	19 (20.2)	7 (10.4)	.10
End-stage Kidney Disease	10 (10.6)	5 (7.5)	.49
Coronary Artery Disease	12 (12.8)	10 (14.9)	.69
Congestive Heart Failure	25 (26.6)	11 (16.4)	.13
Hypertension	31 (33.0)	14 (20.9)	.09
Diabetes	41 (43.6)	21 (31.3)	.12
Chronic lung disease	8 (8.5)	9 (13.4)	.32
Liver disease (severe)	9 (9.6)	4 (6.0)	.41
Metastatic Cancer	1 (1.1)	5 (7.5)	**.04**
Cerebrovascular disease	9 (9.6)	4 (6.0)	.41
Transplant	5 (5.3)	2 (3.0)	.47
Etiology of Acute Kidney Injury⁂			
Sepsis	48 (51.1)	34 (50.7)	.97
Cardiac-surgery Associated	8 (8.5)	3 (4.5)	.32
Non-cardiac Surgery	13 (13.8)	5 (7.5)	.21
Liver-associated	7 (7.4)	3 (4.5)	.44
Malignancy	0 (0)	4 (6.0)	**.02**
Cardiorenal Syndrome	8 (8.5)	6 (9.0)	.92
Nephrotoxin	0 (0)	2 (3.0)	.09
Vasculitis	1 (1.1)	1 (1.5)	.81
Other	21 (22.3)	16 (23.9)	.82
Reason for CKRT Initiation⁂			
Hyperkalemia	26 (27.7)	26 (38.8)	.14
Intoxication	1 (1.1)	2 (3.0)	.37
Volume Overload	58 (61.7)	37 (55.2)	.41
Uremia	3 (3.2)	3 (4.5)	.67
Other	6 (6.4)	6 (9.0)	.54
ICU Location			.48
Medical ICU	43 (45.7)	37 (55.2)	
Cardiovascular ICU	29 (30.9)	18 (26.5)	
Surgical ICU	22 (23.4)	12 (17.6)	
Anticoagulation			.76
Citrate only	52 (55.3)	34 (50.7)	
Heparin only	16 (17.0)	16 (23.9)	
Dual	14 (14.9)	9 (13.4)	
None	12 (12.8)	8 (11.9)	
COVID-19 Positive	26 (27.7)	21 (31.3)	.78
SOFA Score	14.5 (2.9)	13.9 (3.4)	.22

P-values < 0.05 are **Bolded**.

*Values are frequency and column percents unless otherwise specified.

⁂ Patients could be included in more than one category if appropriate, giving values >100%.

### Outcomes (primary analysis)

Primary outcomes are summarized in Table 2. Median and interquartile values in the intention-to-treat analysis for pre-filter CVVH and CVVHD were 21.8 (11.4–45.3) hours and 26.6 (13.0–63.5) hours, respectively (p = 0.02). Mean and standard deviation values were 32.1 ± 28.1 and 40.0 ± 34.2, respectively (p = .002). In the per protocol groups, median and interquartile values for pre-filter CVVH and CVVHD were 24.6 (12.2–45.8) hours and 27.3 (13.1–63.7) hours, respectively (p = 0.06). Mean and standard deviation values were 33.6 ± 28.5 and 40.6 ± 34.4, respectively (p = .01).

**Table 2:** Primary and secondary outcomes by modality (intention-to-treat analysis unless otherwise specified).

*Unadjusted Outcomes* *	*CVVH (n = 94*, *346)*	*CVVHD (n = 67*, *245)*	*P value*
	*N (%)*	*N (%)*	
Filter Life, hours, median (IQR)	21.8 (11.4–45.3)	26.6 (13.0–63.5)	**.02**
Filter Life, hours, mean (SD)	32.1 (28.1)	40.0 (34.2)	**.002**
Number of filters, median (IQR)	2 (1–4)	2 (1–5)	.86
CKRT Days, median (IQR)	3 (2–7)	4 (2–9.25)	.31
Filter Life >72 hours	41 (11.8)	52 (21.2)	**.002**
In-hospital mortality	57 (60.6)	47 (70.1)	.21
Reason for Filter Loss‡			**.04**
Clotting/Clogging	92 (26.7)	43 (17.5)	
Maximum Filter Life	14 (4.1)	22 (8.9)	
Imaging/Procedure/Operating Room	55 (15.9)	39 (15.9)	
Access or Machine Issue	41 (11.9)	36 (14.6)	
Death/Hospice/Transition to iHD	78 (31.9)	54 (32.6)	
Other/Unknown	65 (18.8)	52 (21.1)	
*Modeled Outcomes*	*Mean Ratio (95% CI)*		*P value*
Intention-to-Treat	0.79 (0.65–0.97)		**.02**
Per Protocol	0.82 (0.68–1.00)		**.04**

P-values < 0.05 are **Bolded**.

*Values are frequency and column percents unless otherwise specified.

**Fig 2:**
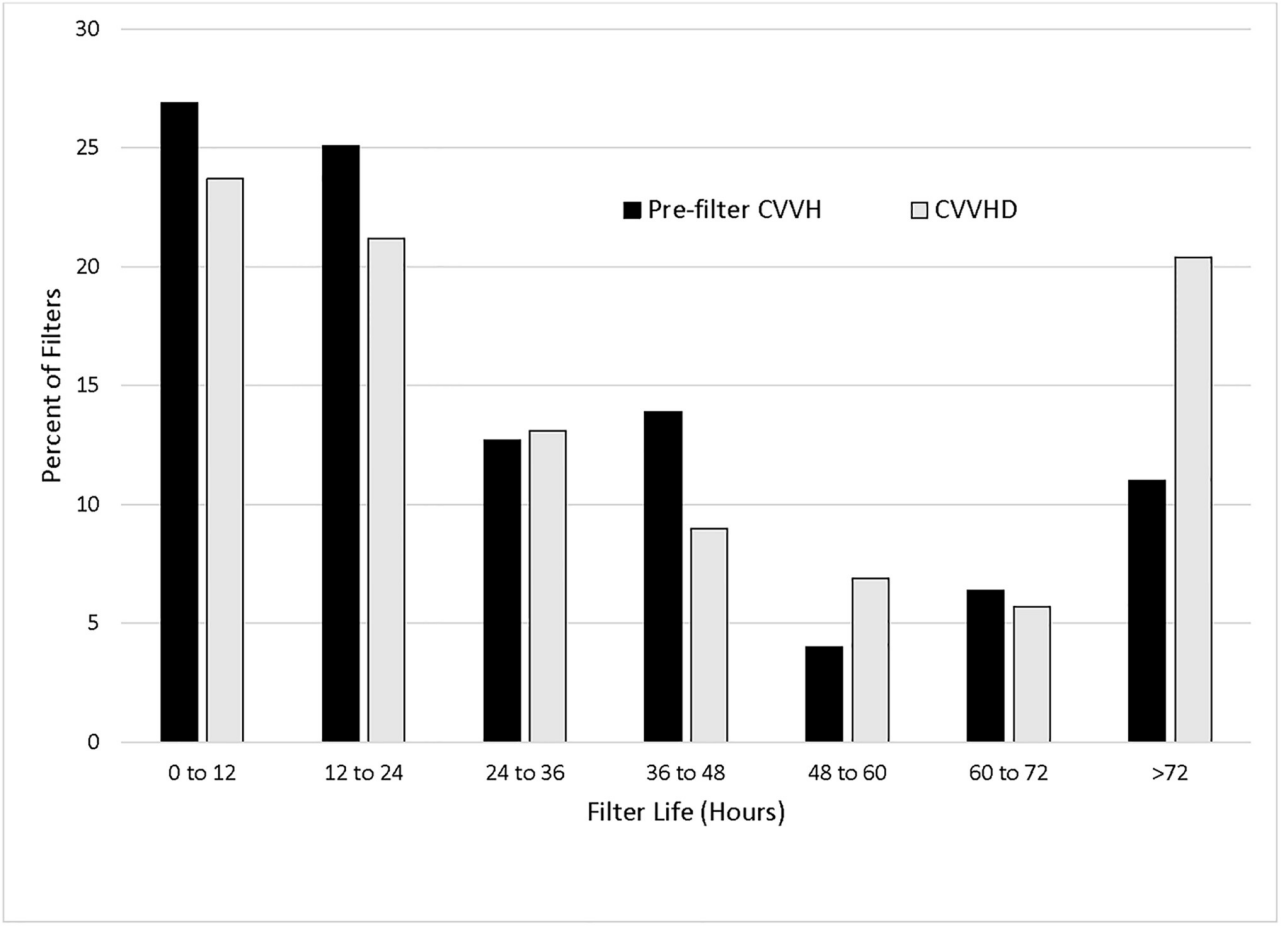
Histogram showing the percentage of filters by filter life category in pre-filter CVVH vs. CVVHD (intention to treat analysis). CVVH–Continuous venovenous hemofiltration; CVVHD–Continuous venovenous hemodialysis.

The median number of filters per patient was 2 in both groups (p = .99). Mortality rates were 60.6% for pre-filter CVVH, and 70.1% for CVVHD, a difference that was not statistically significant (OR 1.5, p = 0.21). In the ITT analysis, 11.8% of pre-filter CVVH and 21.2% of CVVHD filters reached at least 72 hours (OR 2.0, p = .002) (Fig 2). There was also a statistically significant difference in percentage of clotted filters in pre-filter CVVH and CVVHD groups, at 26.7% and 17.5% respectively (p = 0.008).

**Fig 3:**
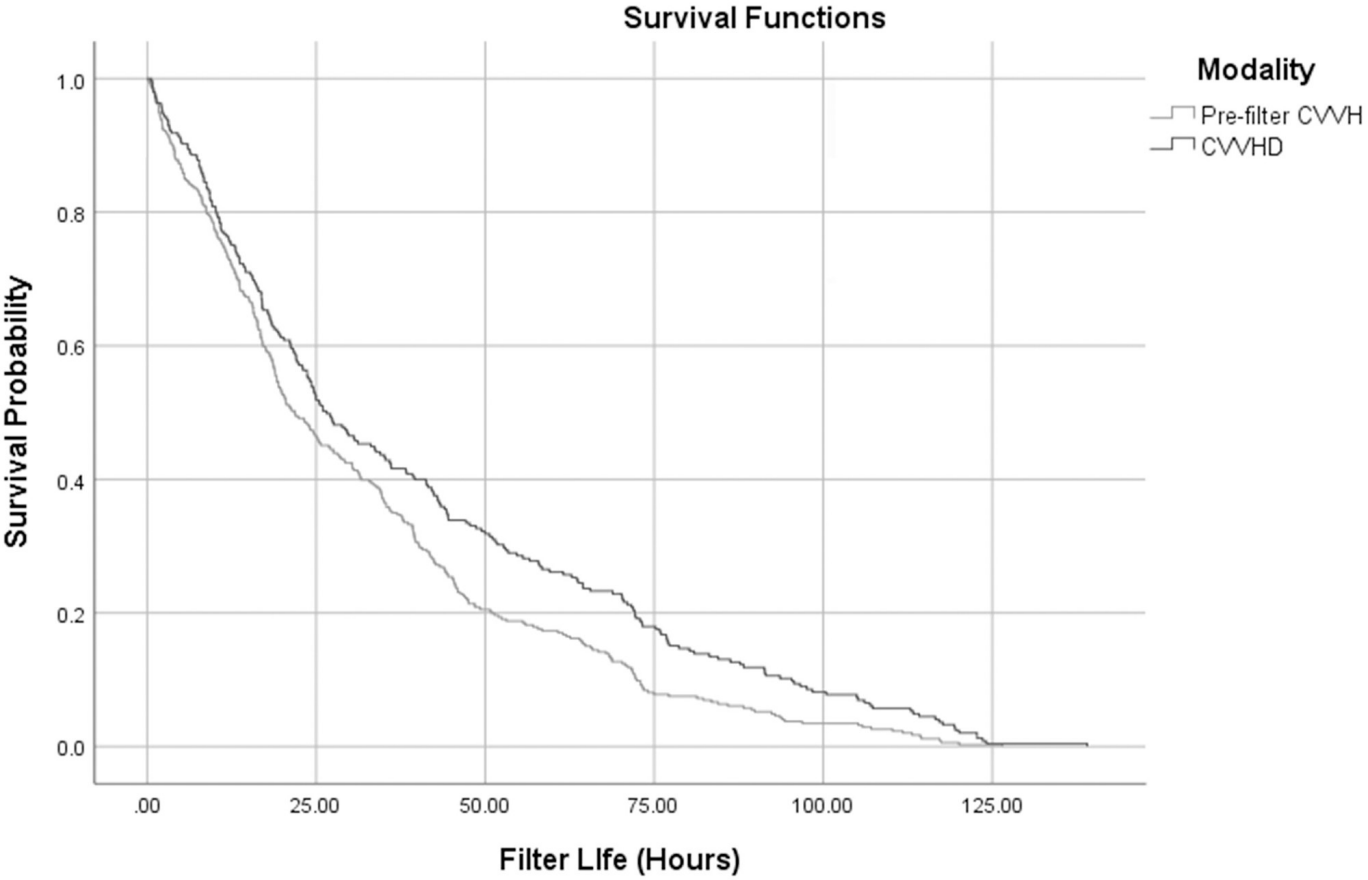
Survival curve of cumulative probability of filter loss in pre-filter CVVH and CVVHD (intention to treat analysis). CVVH–Continuous venovenous hemofiltration; CVVHD–Continuous venovenous hemodialysis.

Statistical modeling as described in the Methods was done to account for multiple filter observations from each patient. In the intention to treat analysis, the mean ratio of filter life for pre-filter CVVH compared to CVVHD was 0.79 (95% CI 0.65–0.97, p = 0.02), which is the ratio of mean filter life in CVVH over CVVHD, adjusted for covariates. In the per protocol analysis, the mean ratio of filter life was 0.82 (0.68–1.00, p = 0.04) (Table 2). A survival curve for filter life is given in Fig 3.

### Outcomes (COVID-19 analysis)

There were a total of 204 filters from 47 patient-sessions with COVID-19, 370 filters from 108 patient-sessions who tested negative for COVID-19, and 17 filters from 6 patient-sessions not tested. After removing filters from untested patients, median and interquartile values in this secondary analysis were 22.7 (10.3–56.8) hours and 24.5 (12.0–47.5) hours, respectively, for COVID-19 and non-COVID-19 filters (p = 0.78). In-hospital mortality rates were 76.6% (36 out of 47) for COVID-19 patients, and 57.4% (62 out of 108) for non-COVID-19 patients (p = 0.02).

### Anticoagulation and COVID-19 interactions

For anticoagulation, the interaction p-value was marginal at 0.09, and when stratified into heparin-only and citrate-only, the interaction p-values were 0.07 and 0.15, respectively. The COVID interaction p-value was 0.32.

## Discussion

In this quasi-randomized pragmatic cluster trial of critically ill patients requiring CKRT, we found that filter life in pre-filter CVVH was 79% of filter life in CVVHD, a result that was statistically significant. The median absolute difference was 4.8 hours, and the mean absolute difference was 7.9 hours. The percentage of filters changed due to clotting was higher (26.7%) for the pre-filter CVVH group than for CVVHD group (17.5%). Finally, a total of 11.8% of pre-filter CVVH vs 21.2% in CVVHD reached 72 hours, suggesting that the differences in filter life were driven in part by the number of filters reaching maximum life.

Filter loss is a major problem in CKRT delivery which results in a decrease in time on treatment, reductions in daily delivered effluent dose, and blood loss. In addition, more frequent filter changes increase the cost of CKRT delivery [3]. The average patient is on CKRT for 3–7 days [14], and sometimes substantially longer, so even modest differences in filter life can be impactful. Downtime has been reported to be as high as 3 hours per day on average [15]. As a result, filter life has been identified as a key CKRT quality metric and has been the subject of several quality improvement initiatives [4].

Circuit clotting can occur for a variety of reasons, including suboptimal vascular access, inadequate anticoagulation, or underlying patient factors. One possible contributing factor that has been relatively understudied is CKRT modality, and in fact, few previous studies have evaluated CKRT modality as a risk factor for filter loss. In one study from 2006, Ricci et al. evaluated differences in solute removal between CVVH and CVVHD in 15 patients, and found that CVVHD had a significantly longer half-life (37 hours vs 19 hours) [16]. However, this study used a roughly 50–50 mix of pre-filter and post-filter fluid replacement. Another study of 31 patients with a cross-over design compared high-volume pre-filter CVVH to CVVHDF, and found that filter life was longer in the CVVHDF group, although both groups had very low filter life (16 hours vs 6.5 hours) [10]. A meta-analysis from 2012 including these two studies, plus others comparing filter life at differing CKRT doses, suggested an absolute mean difference of about 5.6 hours between modalities, similar to our findings, albeit with significant heterogeneity [11]. Finally, in a 2020 analysis of 638 patients evaluating filter life with citrate compared to heparin, a post-hoc analysis showed that CVVHD had the longest filter life, followed by CVVHDF and CVVH, which had the worst filter-life [12]. However, the percentage of pre-filter dilution in the CVVH arm was not specified. While these studies all point to longer filter-life in diffusion-based modalities, there were no clear comparisons of pre-filter CVVH to CVVHD.

Our finding of longer filter life in CVVHD can potentially be explained by modality-specific differences in hemoconcentration and concentration shifts within the filter. One major difference between convective (CVVH) modalities using post-filter replacement fluid and diffusive (CVVHD) modalities in seen in filtration fraction (FF), which is the ratio of effluent flow rate divided by plasma flow rate, is a measure of the degree of hemoconcentration that occurs as blood passes through a CKRT filter [17]. Higher rates of ultrafiltration and fluid removal across the filter result in more concentrated blood at the end of the filter, theoretically predisposing to increased clotting. A FF <20% is advocated in order to reduce clotting, and can be achieved by decreasing Q_R_ or increasing the blood flow rate.

Prefilter administration of replacement fluid dilutes blood entering the filter, and therefore prolongs filter life [7]. For example, in a study of 48 patients, median filter life was 5 hours shorter in the post-dilution group compared to pre-dilution (p = 0.021) [7, 15]. Recently, however, the validity of filtration fraction in pre-filter CVVH has been called into question, primarily due to lack of clinical data although also on theoretical grounds [8]. Drs. Hatamizadeh, Tolwani, and Palevsky recently wrote regarding pre-filter CVVH that “it does not change end-of-hemofilter Hct” and therefore “should not affect the likelihood of filter clotting.” However, clinical data to support this assertion are lacking, as these authors note, and in our opinion it is still plausible that even though the final concentrations are unchanged, the rapid shifts in hematocrit and plasma protein concentrations as replacement fluid is quickly added and then removed may increase the likelihood of filter clotting, and may account for the observed findings in this study.

In CVVHD, there is no movement of fluid across the filter other than minor contributions from ultrafiltration, because clearance is achieved through diffusion rather than convection. As a result, there is neither hemoconcentration nor rapid shifting in hematocrit concentration in CVVHD, which may theoretically reduce the rate of filter clotting. This prospective study did show prolonged filter life and reduced filter clotting with CVVHD rather than pre-filter CVVH, as we hypothesized given differences in FF and concentration shifts between the two modalities. Further research into the possible mechanisms of filter clotting by modality is needed.

Our findings have potential implications for CKRT modality selection. Historically, convective clearance or a combination of convection and diffusion has been preferred over diffusion alone. Convection is known to have better middle-molecule clearance than diffusion [16], and so in the past it was hypothesized that removal of cytokines, interleukins, and other mediators of inflammation would improve outcomes in sepsis and other high-inflammation conditions [18]. While there are emerging data that suggest some benefit to middle molecule clearance in patients with end-stage kidney disease [19], multiple studies have failed to demonstrate clinical benefit from higher middle molecule clearance in the AKI population [9, 18, 20].

Pre-filter CVVH is known to have less efficient clearance compared to either CVVHD or post-filter CVVH because blood is diluted prior to entering the circuit. This disadvantage was thought to be off-set by better filter life due to hemodilution of blood before entering the filter [21], and better middle molecule clearance as above. In this study, we found that convective clearance, even when replacement was purely pre-filter, results in shorter filter life compared to diffusion. CVVHD therefore uses less fluid to achieve the same urea clearance and has longer filter-life, so in the absence of proven benefit related to middle-molecule clearance in the acute setting, providers should consider using pure diffusion or diffusion predominant CKRT modalities.

Our trial has multiple limitations that must be acknowledged. The median filter lives in this study of 21.8 and 26.6 hours (32.1 and 40.0 mean hours) for pre-filter CVVH and CVVHD respectively are lower than many, though not all, other published studies using citrate [12, 22]. While the shorter filter lives in this study could indicate an error with existing study citrate protocols, we think a large part of the difference can be explained by differences in patient population. For instance, in a recent comparison of filter life in regional citrate vs. heparin anticoagulation, the median filter life for citrate was 47 hours. However, the study excluded patients with coagulopathies, thrombocytopenia, severe liver disease, severe lactic acidosis, and gastrointestinal bleeds [12], while our study included all these patients.

In addition, COVID-19 affected nearly a third of the AKI-CKRT population in this trial. COVID-19 is known to increase filter clotting [23]. Similarly, the in-hospital mortality rate of 66%, higher than national averages [24], is likely attributable to the onset of COVID-19, which had a mortality rate of 75% in this study. While the rates of COVID-19 infection appear to be balanced between groups, it is possible that the high incidence of COVID-19 infection limits generalizability. Of note, we did not observe significant differences in filter life in COVID compared to non-COVID patients, possibly due to the aggressive use of dual-anticoagulation strategies at UIHC in COVID-positive patients. Additional statistical analysis evaluating interactions between both anticoagulation strategies and COVID-19 status were not significant, suggesting that these variables did not have a significant differential group effect within the models.

Another limitation is that patients were able to change modality during the study at the discretion of the attending on service, which was deemed necessary to ensure that patient risk would be minimal in the study. Modality changes from pre-filter CVVH to CVVHD, and vice-versa, were rare, without a clear directional bias. The most common modality change was from CVVH to CVVHDF, which affected 47 filters, and may have been driven by higher filter pressures in the pre-filter CVVH group. Conversely, changing from CVVHD to CVVHDF was seen in only 17 filters. The per-protocol analysis, however, showed results that were similar to the ITT analysis. Finally, an accidental block repeat at blocks 9 and 10 resulted in 11 pre-filter CVVH blocks compared to 9 CVVHD blocks.

## Conclusion

In this pragmatic trial involving critically ill adults with severe AKI requiring CKRT, filter life was prolonged with use of CVVHD compared to pre-filter CVVH. This finding may lead providers to reduce their use of convective clearance when prescribing CKRT, but further studies from other centers and other populations are needed to validate our results.

## Supporting information

S1 Checklist CONSORT 2010 checklist of information to include when reporting a randomised trial*. (DOCX)

S1 File HawkIRB protocol. (DOCX)

S1 Dataset (PDF)
