# Correction: CVVHD results in longer filter life than pre-filter CVVH: Results of a quasi-randomized clinical trial

**DOI:** 10.1371/journal.pone.0306216

**Published:** 2024-06-21

**Authors:** Lewis Mann, Patrick Ten Eyck, Chaorong Wu, Maria Story, Sree Jenigiri, Jayesh Patel, Iiro Honkanen, Kandi O’Connor, Janis Tener, Meenakshi Sambharia, Mony Fraer, Lama Noureddine, Douglas Somers, Jonathan Nizar, Lisa Antes, Sarat Kuppachi, Melissa Swee, Elizabeth Kuo, Chou-Long Huang, Diana I. Jalal, Benjamin R. Griffin

The 12^th^ author’s name is spelled incorrectly. The correct name is: Lama Noureddine.
