# Supplementary material for: CVVHD results in longer filter life than pre-filter CVVH: Results of a quasi-randomized clinical trial
Source: PLoS One. 2023 Jan 11;18(1):e0278550. doi: 10.1371/journal.pone.0278550 (PMC9833553; doi:10.1371/journal.pone.0278550)
Supplement: S1 File — (DOCX) [file pone.0278550.s002.docx]

**HawkIRB Protocol**

**Project Title: The impact of CRRT modality on filter life**

**Principal Investigator: Dr. Benjamin Griffin**

**Version Date: September 5, 2019**

**Protocol #: 18-0405**

**Project Title: Metabolomic Changes Following Initiation of Continuous Renal Replacement Therapy**

**Principal Investigator: Dr. Benjamin Griffin**

**Version Date: August 19, 2019**

1. **Hypotheses and Specific Aims**:

**Research Question: Does the choice of modality (CVVH vs CVVHD) impact filter life in CRRT?**

**Specific Aim 1: Determine the average filter life in patients initiated on CVVH and CVVHD.** In this pilot study, we plan to enroll 250 patients with severe AKI who are being initiated on CRRT, and estimate a total of 1,000 filters will be used. Patients will be cluster-randomized based on date to initiate CRRT with either pre-filter CVVH or CVVHD, with the primary outcome of average filter life.

1. **Background and Significance**:

Severe acute kidney injury (AKI) requiring dialysis has an in-hospital mortality rate > 50%, making it one of the deadliest patient conditions commonly encountered in US hospitals^1^. In patients with AKI, dialysis – also known as renal replacement therapy (RRT) – is the only FDA approved therapy. Continuous RRT (CRRT) is generally the preferred option for RRT in the ICU because it is associated with less hemodynamic instability than intermittent therapy^2^.

Filter loss due to clotting is a common problem on CRRT which reduces the patient’s time receiving therapy and increases cost of care. Even with the use of citrate anticoagulation, average filter life is less than 40 hours in reported literature^3,4^.

There are many factors involved in filter clotting, including access location, anticoagulation use, and factors related to the patient’s illness^5^. An additional factor that is frequently advanced is filtration fraction. Filtration fraction is a measure of the degree of hemoconcentration through a CRRT filter. Higher rates of ultrafiltration and fluid removal across the filter result in more concentrated blood at the end of the filter, theoretically predisposing to increased clotting. The filteration fraction calculation in post-filter CVVH is as follows^6^:

$$FF= \frac{Q_{R}+UF}{Q_{BW}}$$

Where Q_R_ is the Replacement fluid flow rate (L/hr), UF is the amount of ultrafiltration (L/hr), and Q_BW_ is the blood flow rate (BFR) in L/hr * (1 – hematocrit). A filtration fraction <20% is advocated in order to reduce clotting, and can be achieved by decreasing Q_R_ or increasing BFR.

Prefilter administration of replacement fluid decreases the solute concentration in the blood entering the filter, and therefore prolongs filter life. The use of dialysate as opposed to replacement fluid results in no hemoconcentration at all, as diffusion rather than convection is the method of clearance. It is therefore unclear which modality better decreases the potential for filter clotting, and so it is currently unclear whether there is a difference in filter life between these two modalities. The purpose of this study iss to examine filter life in patients treated with either pre-filter CVVH or CVVHD, with the hypothesis that filter life would be longer in patients treated with CVVHD as opposed to pre-filter CVVH.

**III**. **Preliminary Studies/Progress Report:**

No published studies were found to determine whether pre-filter CVVH or CVVHD prolong filter life. An abstract presented at American Society of Nephrology Kidney Week 2018 showed in a retrospective study that pre-filter CVVHD had shorter filter life, with a HR of 1.3, but these results were not published.

**IV. Research** **Methods**

**A. Outcome Measure(s):**

**Primary Outcomes:**

- Time to filter loss (all filters)
- Average filter life (for all filters, adjusted for multiple events within the same patient)

**Secondary Outcomes:**

- Time to first filter loss, censored for mortality, or interrupting events such as operating room visits)
- Time to death (censored for patient drop out or loss to follow up)
- Mortality at 60 days
- Time to renal recovery (censored for interfering events)
- Renal Recovery at 60 days
- RRT free days at 60 days
- ICU-free days at 60 days
- Hospital-free days at 60 days

1. **Description of Population to be Enrolled:**

**Population**

- Adult patients initiated on CRRT at UIHC

**Exclusion Criteria**

- ECMO use at the time of CRRT initiation
- Inability to safely receive citrate or heparin anticoagulation

1. **Study Design and Research Methods**

**Trial Design**

- **Randomization:**
  - Cluster randomization to either pre-filter CVVH or CVVHD depending on month of the year.
    - Patients initiated on odd months will receive CVVH, and patients initiated during even months will receive CVVHD.
- All patients will be prescribed an initial BFR of 200 mL/min and an initial dose of 30 mL/kg. Ultrafiltration will be prescribed by the renal team based on clinical needs. Adjustments to the fluid flow rates will be at the discretion of team, with a recommended delivered dose 20-30 mL/kg/hr. Filter changes will be made at the discretion of the nurse managing CRRT, as part of standard practice at UIHC. Filters will be changed at least every 72 hours, as is standard at UIHC. Citrate will be used in all patients, as is standard at UIHC. All patients will have a post-filter fluid replacement of 200 mL/hour, as recommended for Prismaflex machines.

**Variables to be collected:**

- Demographic data (Age, Gender, Race, BMI)
- SOFA score at initiation
- Comorbidities
- Cause of AKI (Sepsis, post-surgical, etc.) AND/OR location of ICU (medical vs surgical)
- Laboratory data at baseline (BMP, CBC, lactate, albumin, INR)
- Daily laboratory data
- % Volume overload at initiation and daily
- HD catheter location
- CRRT variables (BFR, dose, therapy fluid flow rate, UF, filtration fraction).
- Reason for filter loss.

**Power calculations:**

- Using a power of 0.8 and an alpha of 5%
- Previous data suggest a hazard ratio of 1.3. Estimated overall probability of filter loss due to clotting (as opposed to end-of-therapy, e.g) of 60%. Expected 50% of patients in each group.
- Total number of filters needed is 1,010. Given an expected average of 4 filters per patient over the course of CRRT, 253 patients are needed.
- UIHC initiates 1,100 circuits per year, on average. Therefore it is estimated that enrollment will be completed over 1 year.

**D. Description, Risks and Justification of Procedures and Data Collection Tools:**

**Justificiation of minimal risk:** These patients are being initiated on CRRT for clinical rather than research reasons. There is currently thought to be equipoise between modalities within CRRT. The patient will therefore not be at increased risk. We therefore believe that a cluster-randomized study will not adversely affect the subject's rights or welfare.

**Human subject protection**: We are requesting a waiver of consent for this study. CRRT is often initiated emergently in critically ill patients due to life-threatening metabolic derangements. More than 80% of patients are intubated and sedated at the time of initiation. Because CRRT is often started emergently, there is often inadequate time to obtain consent from a legally authorized representative. Currently, modalities of CRRT are thought to be equivalent, and no study has shown definitively that use of convective versus diffusive clearance is of any benefit. Decisions about modality are currently based on provider preference, and often include elements of both types of clearance (CVVHDF). Because of the equipoise among modalities, we feel that this study is minimal risk, and the randomization strategy (cluster randomization) and difficulties in obtaining consent outlined above warrant a waiver of consent.

**E. Potential Scientific Problems:**

Possible problems include:

1. Inability to recruit sufficient patients.
   1. Given that an estimated 1100 circuits were used last year, we expect to reach a target of 1,000 filters (based on power calculations) within one year.
2. Inability to obtain consent
   1. Due to the difficulties with consent in population, we believe this study would be impracticable without a waiver of consent, and could also introduce selection bias. We are therefore requesting a waiver of consent.

**F. Data Analysis Plan:**

**Brief Statistical Analysis Plan:**

Average filter life will compared between groups using means and t-tests. Time to filter loss will be evaluated between the groups using Cox proportional hazard modeling, with an analysis of competing risks to censor for mortality. Comparisons between exposures for RRT-free, ICU-free and hospital-free days at 60 days will be made using Poisson regression.

**G. Summarize Knowledge to be Gained:**

In this proposal, ***we expect*** to determine whether pre-filter CVVH or CVVHD have longer average filter lives, or if there is indeed equipoise as currently thought. We expect this data to assist in decisions of modality in the future. In addition, we will be able to retrospectively determine the relative contributions of other known factors in filter life, such as access location, type of anticoagulation, and factors related to the patient’s illness, which may lead to extended filter live and improved quality of care in the future.

**H. References**

1. Iwagami M, Yasunaga H, Noiri E, et al. Current state of continuous renal replacement therapy for acute kidney injury in Japanese intensive care units in 2011: analysis of a national administrative database. Nephrology, dialysis, transplantation : official publication of the European Dialysis and Transplant Association - European Renal Association 2015;30:988-95.

2. Deepa C, Muralidhar K. Renal replacement therapy in ICU. Journal of anaesthesiology, clinical pharmacology 2012;28:386-96.

3. Stucker F, Ponte B, Tataw J, et al. Efficacy and safety of citrate-based anticoagulation compared to heparin in patients with acute kidney injury requiring continuous renal replacement therapy: a randomized controlled trial. Crit Care 2015;19:91.

4. Gutierrez-Bernays D, Ostwald M, Anstey C, Campbell V. Transition From Heparin to Citrate Anticoagulation for Continuous Renal Replacement Therapy: Safety, Efficiency, and Cost. Ther Apher Dial 2016;20:53-9.

5. Brain M, Winson E, Roodenburg O, McNeil J. Non anti-coagulant factors associated with filter life in continuous renal replacement therapy (CRRT): a systematic review and meta-analysis. BMC Nephrol 2017;18:69.

6. Macedo E, Mehta RL. Continuous Dialysis Therapies: Core Curriculum 2016. Am J Kidney Dis 2016;68:645-57.
