# Supplementary material for: CVVHD results in longer filter life than pre-filter CVVH: Results of a quasi-randomized clinical trial
Source: PLoS One. 2023 Jan 11;18(1):e0278550. doi: 10.1371/journal.pone.0278550 (PMC9833553; doi:10.1371/journal.pone.0278550)
Supplement: S1 Dataset — (PDF) [file pone.0278550.s003.pdf]

Patient ID	Filter Number	Filter Life (hours)	CRRT Modality (Intention To Treat)
1	1	72.2	0
1	2	49.27	0
2	1	68.73	0
3	2	7.02	0
3	1	34.98	0
4	1	5	0
5	1	15.73	0
6	1	72.45	0
7	1	13.05	1
7	2	17.02	1
7	3	29.75	1
7	4	3.35	1
8	1	23.03	1
8	2	71.67	1
8	3	9.38	1
8	4	9.25	1
8	5	33.65	1
8	6	8.07	1
8	7	0.8	1
8	8	8.8	1
8	9	23.67	1
8	10	2.07	1
8	11	41.33	1
8	12	25.42	1
9	1	3.13	1
9	2	5	1
10	1	72.13	1
10	2	9.25	1
11	1	4.55	1
11	3	12.38	1
11	4	13.6	1
11	2	21.57	1
12	1	1.23	1
12	2	17.05	1
12	3	31.17	1
12	4	29.62	1
12	5	33.8	1
12	6	10.88	1
12	7	17.95	1
12	8	7.4	1
12	12	10.77	1
12	9	13.33	1
12	10	7.73	1
12	11	75.25	1
12	13	41.23	1
12	14	44.42	1

12	15	8.93	1
13	1	72.27	1
14	1	73.25	1
15	1	78.63	1
15	2	24.57	1
16	1	72.9	1
16	2	70.33	1
16	3	120.08	1
16	4	88.25	1
16	5	106.93	1
16	6	87.72	1
17	1	34.53	0
17	2	23.17	0
17	3	64.82	0
17	4	31.37	0
17	5	18.32	0
17	6	43.92	0
17	7	16.78	0
17	8	30.53	0
17	9	82.27	0
17	11	16.77	0
17	12	41.97	0
17	14	114.35	0
17	15	17.6	0
17	16	74.95	0
17	17	19.22	0
17	18	17.07	0
17	13	20.28	0
17	10	71.12	0
18	2	74.07	0
18	1		
19	1	1.18	0
19	2	7.68	0
19	3	9.88	0
19	4	15.7	0
19	5	5.07	0
19	6	1.53	0
20	1	2.25	0
21	1	40.67	0
21	2	117.47	0
22	1	37.58	0
22	2	13.1	0
22	3	107.1	0
23	1	21.87	0
23	2	30.4	0
23	3	68.33	0
24	3	44.52	0

24	1	42.2	0
24	2	25.33	0
25	1	70.43	1
25	2	24.83	1
25	3	24.65	1
25	4	42.97	1
25	5	63.53	1
26	4	68.43	0
26	5	42.47	0
26	1	83.45	0
26	2	47.52	0
26	3	19.15	0
27	1	24	0
27	2	25.5	0
27	3	16.37	0
28	1	23.67	1
29	1	18.5	1
29	10	91.37	1
29	11	122.75	1
29	2	35.9	1
29	3	83.17	1
29	4	95.33	1
29	5	63.77	1
29	6	58.08	1
29	7	59.5	1
29	8	26.03	1
29	9	91.33	1
30	1	80.23	1
30	2	16.27	1
31	1	9.23	1
32	8	34.73	0
32	9	38.62	0
32	10	17.08	0
32	1	10.95	0
32	2	34	0
32	3	12.4	0
32	4	44.15	0
32	5	24.5	0
32	6	20.05	0
32	13	45.28	0
32	14	65.88	0
32	15	14.17	0
32	16	50.97	0
32	19	94.32	0
32	21	64	0
32	7	47.48	0
32	11	20.38	0

32	12	72.25	0
32	17	21.22	0
32	18	39.68	0
32	20	71.65	0
33	2	0.68	0
33	1	5.25	0
33	3	16.28	0
33	4	12.77	0
33	5	0.47	0
33	6	1.68	0
33	16	2.18	0
33	19	57.07	0
33	20	21.8	0
33	7	24.78	0
33	8	27.03	0
33	9	27.55	0
33	10	15.55	0
33	11	13.57	0
33	12	13.27	0
33	13	0.53	0
33	14	105.17	0
33	15	10.32	0
33	17	15.5	0
33	18	31.62	0
34	1	23.92	0
34	2	26.92	0
34	3	2.8	0
34	4	105.83	0
34	5	93.58	0
34	6	24.63	0
35	1	34.58	0
36	1	29.13	0
36	2	12.58	0
37	1	0.37	0
38	1	4.6	0
39	1	3.22	1
39	2	124.17	1
39	3	96.6	1
40	1	3.12	1
41	1	0.92	1
41	2	55.58	1
41	3	119.5	1
41	4	5.1	1
41	5	20.18	1
42	3	76.95	1
42	4	68.87	1
42	5	22	1

42	1	1.35	1
42	2	7.3	1
42	6	139.05	1
42	7	25	1
42	8	117.13	1
42	9	112.92	1
42	10	117.68	1
42	11	51.5	1
43	1	13.65	1
43	2	30.72	1
44	1	6	1
44	2	0.77	1
45	1	8.3	1
45	2	11.68	1
45	3	16.92	1
46	4	50.67	1
46	5	12.17	1
46	6	6.45	1
46	7	15.67	1
46	8	44.55	1
46	1	35.18	1
46	2	29.33	1
46	3	113.32	1
47	1	10.5	1
48	1	64.45	1
49	1	5.48	0
49	2	19.43	0
49	3	45.97	0
50	1	96.88	0
50	2	16.77	0
51	1	51.42	0
52	1	16.8	0
52	2	47.35	0
52	3	126.5	0
52	4	19.45	0
53	1	62.78	1
53	2	42.93	1
54	1	76.77	1
54	2	5.97	1
54	3	77.22	1
55	1	11.35	1
56	1	64.42	1
56	2	27.1	1
56	3	56.57	1
56	4	19.77	1
56	5	43.35	1
56	6	19.25	1

56	7	21.28	1
57	1	36.12	1
58	4	22.92	1
58	5	100.42	1
58	1	2.5	1
58	2	14.25	1
58	3	22.05	1
59	1	107.3	1
59	2	47.73	1
60	1	20.5	0
60	2	16.97	0
60	3	16.18	0
60	4	3.5	0
60	5	39.47	0
60	6	64.42	0
61	1	61.95	0
61	2	66.75	0
61	3	13.68	0
61	4	13.75	0
62	1	93.9	0
63	1	65.6	0
63	2	55.92	0
63	3	45.53	0
63	4	6.25	0
64	1	111.75	0
64	2	45.18	0
65	1	110.25	0
65	2	73.15	0
65	3	81.3	0
66	4	39.27	0
66	5	11.37	0
66	6	40.3	0
66	7	21.3	0
66	1	37	0
66	2	3.45	0
66	3	57.75	0
67	1	19.87	0
67	2	39.23	0
68	1	11.6	0
68	2	40.03	0
68	3	5.55	0
68	4	9.47	0
69	1	85.9	0
69	2	50.72	0
70	1	0.07	0
71	1	20.58	0
72	1	5.27	0

73	1	37.8	0
73	2	61.5	0
73	3	5	0
74	1	46.8	0
74	2	22.38	0
74	3	25.23	0
74	4	6.1	0
74	5	9.87	0
74	6	32.92	0
74	7	16.33	0
75	2	39.3	0
75	1	39.5	0
76	1	52.45	0
77	1	18.15	0
77	2	89.42	0
78	1	37.95	0
78	2	51.95	0
78	3	25.68	0
79	1	44.37	0
79	2	12.28	0
80	1	46.33	0
80	2	12.08	0
81	1	68.75	0
81	2	41.38	0
81	3	15.88	0
81	4	89.9	0
81	5	21	0
82	1	2.22	0
82	2	5.75	0
83	1	15	0
84	1	37.83	0
85	1	8.08	0
85	2	18.82	0
86	1	2.22	1
86	2	70.18	1
87	1	24.12	0
89	1	84.45	1
90	1	0	1
91	1	21.18	1
91	2	10.27	1
91	3	51.03	1
91	4	42.05	1
91	5	95.7	1
92	1	38.23	1
93	1	52.7	1
93	2	86.25	1
93	3	114.2	1

94	9	91.47	1
94	10	76.92	1
94	8	21.13	1
94	1	53.38	1
94	2	23.72	1
94	3	18.23	1
94	4	4.83	1
94	5	27.55	1
94	6	16.5	1
94	7	39.25	1
95	1	35.98	1
95	2	54.68	1
95	3	22.1	1
96	4	3.22	0
96	5	2.38	0
96	6	88.2	0
96	1	42.72	0
96	2	33.27	0
96	3	84.47	0
97	1	33.58	0
98	3	18.47	0
98	4	23.28	0
98	1	8.07	0
98	2	8.38	0
99	1	41.77	0
100	1	19.7	0
100	2	117.18	0
100	3	60.77	0
100	4	28.12	0
101	1	34.68	0
101	2	34.38	0
102	1	105.02	1
103	1	11.3	0
103	2	72.03	0
103	3	31.15	0
103	4	45.72	0
104	1	1.68	0
104	2	1.4	0
104	3	1.6	0
104	4	4.2	0
104	5	13.67	0
104	6	35.37	0
105	1	15.18	0
106	1	25.05	0
106	2	23.62	0
106	3	35.37	0
107	1	31.33	0

107	2	17.72	0
107	3	18.93	0
107	4	42.45	0
108	1	13.63	0
108	2	12.97	0
108	3	4.58	0
108	4	18.9	0
109	1	19.15	0
109	2	18.75	0
109	3	8.72	0
110	1	1.82	0
111	1	16.83	1
111	2	27.43	1
111	3	42.27	1
111	4	71.9	1
112	1	18.78	1
112	3	21.78	1
112	6	35.12	1
112	7	16.13	1
112	8	13.1	1
112	9	0.83	1
112	2	24.28	1
112	4	9.62	1
112	5	49.42	1
113	1	105.08	1
114	1	17	1
116	1	1.15	1
116	2	2.32	1
116	3	8.8	1
116	4	44.27	1
116	5	22.4	1
116	6	8.4	1
116	7	10.87	1
116	8	30.55	1
116	9	8.03	1
116	10	7.73	1
116	11	7.8	1
116	12	15.52	1
116	13	1.27	1
116	14	13.68	1
116	15	10.62	1
116	16	17.12	1
117	1	16.87	0
117	2	39.37	0
117	3	53.12	0
118	1	61.55	1
119	1	114.48	0

119	2	11.88	0
120	1	73.35	0
121	1	2.9	0
121	2	11.93	0
121	3	2.02	0
121	4	1	0
121	5	70.68	0
121	6	9.12	0
121	7	20.5	0
122	1	45.95	0
122	2	45.52	0
122	3	28.48	0
122	4	19.83	0
122	5	18.62	0
123	1	39.53	0
123	2	12.95	0
126	1	73.15	1
126	2	16.92	1
128	1	10.78	0
129	1	75.82	1
130	1	48.6	1
131	1	113.13	0
131	2	48.48	0
131	3	71.83	0
132	1	13.62	0
133	1	18.17	1
134	1	16.32	0
135	1	2.88	1
135	2	8.37	1
135	3	44.48	1
135	4	39.55	1
135	5	72.18	1
136	2	14.32	0
136	3	8.17	0
136	1	11.78	0
136	4	9.37	0
136	5	15.08	0
137	1	44.07	0
137	2	1.5	0
138	1	49.95	1
138	2	14.55	1
138	3	2.17	1
138	4	24.03	1
139	1	15.92	0
139	2	1.93	0
139	3	17.13	0
140	1	52.97	1

141	1	71.77	0
142	1	9.6	1
142	2	6.3	1
142	3	28.83	1
142	4	10.92	1
142	5	80.92	1
143	1	1.87	0
143	2	35.12	0
143	3	1.27	0
143	4	70.25	0
143	5	62.57	0
143	6	35.85	0
143	7	15.68	0
143	8	3.75	0
143	9	36.2	0
143	10	43.28	0
143	11	3.92	0
143	12	8.13	0
143	13	31.57	0
143	14	11.05	0
143	15	1.05	0
143	16	2.15	0
143	17	11.57	0
143	18	10.5	0
143	19	30.3	0
143	20	7.87	0
143	21	72.98	0
143	22	120.08	0
143	23	4.12	0
143	24	15.7	0
143	25	18.93	0
143	26	28.6	0
143	27	12.45	0
143	28	34.87	0
144	1	10.1	1
144	2	3.57	1
144	3	25.6	1
144	4	41.52	1
144	5	25.98	1
144	6	15.88	1
144	7	57.87	1
144	8	19.5	1
144	9	24.97	1
145	1	30.32	0
145	2	4.08	0
146	1	12.07	1
147	1	40.2	0

148	1	72.98	0
149	1	73.43	0
149	2	55.58	0
149	3	19.37	0
149	4	4.07	0
149	5	13.3	0
149	6	29.3	0
149	7	9.95	0
149	8	41.72	0
149	9	18.12	0
149	10	5.47	0
150	1	17.87	1
151	1	47	0
151	2	58.37	0
152	3	77.15	0
152	1	18.68	0
152	2	14.45	0
152	4	8.68	0
152	6	64.32	0
152	7	7.63	0
152	8	35.9	0
152	9	45.38	0
152	5	16.42	0
153	1	65.5	1
154	3	27.53	0
154	4	18.52	0
154	5	9.73	0
154	6	6.67	0
154	1	21.98	0
154	2	10.35	0
155	1	9.55	0
155	2	10	0
155	3	39.62	0
155	4	92.33	0
155	9	4.05	0
155	10	2.28	0
155	11	1.7	0
155	5	48.5	0
155	6	8.7	0
155	7	8.82	0
155	8	67.92	0
155	12	3.37	0
155	13	3.13	0
155	14	17.57	0
156	1	92.85	0
157	1	7.42	0
157	2	10.25	0

158	1	43.27	1
158	2	24.28	1
159	1	106.12	1
160	1	98.42	1
160	2	93.6	1
160	3	51.75	1
160	4	27.25	1
160	5	33.08	1
161	1	58.38	1
161	2	119.42	1
161	3	26.57	1
161	4	14.52	1
161	5	12.98	1
161	6	18.42	1
161	7	0.65	1
162	1	10.17	1
163	1	3.97	0
163	2	1	0

CRRT Modality (Per Protocol)	Time Block	COVID-19 Positive	Anticoagulation Group
0	1.00	Not tested	Both
0	1.00	Not tested	Both
0	1.00	Negative	Citrate
0	1.00	Negative	None
0	1.00	Negative	Citrate
0	1.00	Not tested	Citrate
0	1.00	Positive	Heparin
0	1.00	Negative	Citrate
1	2.00	Negative	Citrate
1	2.00	Negative	Citrate
1	2.00	Negative	Citrate
1	2.00	Negative	Citrate
1	2.00	Negative	Heparin
1	2.00	Negative	Heparin
1	2.00	Negative	Heparin
1	2.00	Negative	Heparin
1	2.00	Negative	Heparin
1	2.00	Negative	Heparin
1	2.00	Negative	Heparin
1	2.00	Negative	Heparin
1	2.00	Negative	Heparin
1	2.00	Negative	Heparin
1	2.00	Negative	Heparin
1	2.00	Negative	Heparin
1	2.00	Negative	Citrate
1	2.00	Negative	Citrate
1	2.00	Not tested	Citrate
1	2.00	Not tested	Citrate
1	2.00	Not tested	Heparin
1	2.00	Not tested	Heparin
1	2.00	Not tested	Heparin
1	2.00	Not tested	Both
1	2.00	Positive	Heparin
1	2.00	Positive	Heparin
1	2.00	Positive	Heparin
1	2.00	Positive	Heparin
1	2.00	Positive	Heparin
1	2.00	Positive	Heparin
2	2.00	Positive	Heparin
2	2.00	Positive	Heparin
0	2.00	Positive	Citrate
2	2.00	Positive	Both
2	2.00	Positive	Both
2	2.00	Positive	Both
0	2.00	Positive	Both
0	2.00	Positive	Both

0	2.00	Positive	Both
1	2.00	Negative	Heparin
1	2.00	Negative	Heparin
1	2.00	Negative	Heparin
1	2.00	Negative	Heparin
1	2.00	Positive	Heparin
1	2.00	Positive	Heparin
1	2.00	Positive	Heparin
1	2.00	Positive	Heparin
1	2.00	Positive	Heparin
1	2.00	Positive	Heparin
0	3.00	Negative	Heparin
0	3.00	Negative	Heparin
0	3.00	Negative	Heparin
0	3.00	Negative	Heparin
0	3.00	Negative	Heparin
0	3.00	Negative	Heparin
0	3.00	Negative	Heparin
0	3.00	Negative	Heparin
0	3.00	Negative	Heparin
0	3.00	Negative	Heparin
0	3.00	Negative	Heparin
0	3.00	Negative	Heparin
0	3.00	Negative	Heparin
0	3.00	Negative	Heparin
0	3.00	Negative	Heparin
0	3.00	Negative	Heparin
0	3.00	Negative	Citrate
0	3.00	Negative	Both
0	3.00	Negative	Citrate
	3.00	Negative	None
0	3.00	Positive	Heparin
0	3.00	Positive	Heparin
0	3.00	Positive	Heparin
0	3.00	Positive	Heparin
0	3.00	Positive	Heparin
0	3.00	Positive	Heparin
0	3.00	Negative	Citrate
0	3.00	Positive	Heparin
0	3.00	Positive	Heparin
2	3.00	Positive	Heparin
2	3.00	Positive	Heparin
2	3.00	Positive	Heparin
0	3.00	Negative	Citrate
0	3.00	Negative	Citrate
0	3.00	Negative	Citrate
0	3.00	Not tested	Heparin

0	3.00	Not tested	Citrate
0	3.00	Not tested	Citrate
1	2.00	Not tested	Citrate
1	2.00	Not tested	Citrate
1	2.00	Not tested	Citrate
1	2.00	Not tested	Citrate
1	2.00	Not tested	Citrate
0	3.00	Negative	Heparin
0	3.00	Negative	Heparin
0	3.00	Negative	Both
0	3.00	Negative	Both
0	3.00	Negative	Both
0	3.00	Negative	None
0	3.00	Negative	Citrate
0	3.00	Negative	Citrate
1	4.00	Positive	Heparin
1	4.00	Positive	Heparin
0	4.00	Positive	Heparin
0	4.00	Positive	Citrate
1	4.00	Positive	Both
1	4.00	Positive	Both
1	4.00	Positive	Both
1	4.00	Positive	Both
1	4.00	Positive	Both
1	4.00	Positive	Both
1	4.00	Positive	Both
1	4.00	Positive	Both
1	4.00	Negative	Heparin
1	4.00	Negative	Heparin
1	4.00	Negative	Citrate
0	5.00	Negative	Heparin
0	5.00	Negative	Heparin
0	5.00	Negative	Heparin
0	5.00	Negative	Citrate
0	5.00	Negative	Citrate
0	5.00	Negative	Citrate
0	5.00	Negative	Citrate
0	5.00	Negative	Citrate
0	5.00	Negative	Citrate
0	5.00	Negative	Citrate
0	5.00	Negative	Citrate
0	5.00	Negative	Citrate
0	5.00	Negative	Citrate
0	5.00	Negative	Citrate
0	5.00	Negative	Citrate
0	5.00	Negative	Both
0	5.00	Negative	Both

0	5.00	Negative	Both
0	5.00	Negative	Both
0	5.00	Negative	Both
0	5.00	Negative	Both
0	5.00	Positive	None
0	5.00	Positive	Citrate
0	5.00	Positive	Citrate
0	5.00	Positive	Citrate
0	5.00	Positive	Citrate
0	5.00	Positive	Citrate
1	5.00	Positive	Citrate
1	5.00	Positive	Citrate
1	5.00	Positive	Citrate
0	5.00	Positive	Both
0	5.00	Positive	Both
0	5.00	Positive	Both
0	5.00	Positive	Both
0	5.00	Positive	Both
0	5.00	Positive	Both
0	5.00	Positive	Both
0	5.00	Positive	Both
1	5.00	Positive	Both
1	5.00	Positive	Both
1	5.00	Positive	Both
0	5.00	Positive	Citrate
0	5.00	Positive	Citrate
0	5.00	Positive	Citrate
0	5.00	Positive	Both
0	5.00	Positive	Both
0	5.00	Positive	Both
0	5.00	Negative	Citrate
0	5.00	Negative	Both
0	5.00	Negative	Both
0	5.00	Negative	Citrate
2	5.00	Negative	Heparin
1	6.00	Positive	Both
1	6.00	Positive	Both
1	6.00	Positive	Both
1	6.00	Negative	None
1	4.00	Positive	None
1	4.00	Positive	None
1	4.00	Positive	None
1	4.00	Positive	None
1	4.00	Positive	None
1	6.00	Negative	Heparin
1	6.00	Negative	Heparin
1	6.00	Negative	Heparin

1	6.00	Negative	Citrate
1	6.00	Negative	Citrate
1	6.00	Negative	Both
1	6.00	Negative	Both
1	6.00	Negative	Both
1	6.00	Negative	Both
1	6.00	Negative	Both
1	6.00	Negative	Both
1	6.00	Negative	Citrate
1	6.00	Negative	Citrate
1	6.00	Negative	Citrate
1	6.00	Negative	Citrate
1	6.00	Negative	Heparin
1	6.00	Negative	Heparin
1	6.00	Negative	Heparin
1	6.00	Negative	None
1	6.00	Negative	None
1	6.00	Negative	None
1	6.00	Negative	None
1	6.00	Negative	None
1	6.00	Negative	Citrate
1	6.00	Negative	Citrate
1	6.00	Negative	Citrate
1	6.00	Negative	Citrate
1	6.00	Negative	Citrate
0	7.00	Negative	Citrate
0	7.00	Negative	Citrate
0	7.00	Negative	Citrate
0	7.00	Negative	Both
0	7.00	Negative	Both
0	7.00	Negative	Citrate
2	7.00	Positive	Both
2	7.00	Positive	Both
0	7.00	Positive	Both
0	7.00	Positive	Both
1	8.00	Negative	Citrate
1	8.00	Negative	Citrate
1	8.00	Positive	Citrate
1	8.00	Positive	Both
1	8.00	Positive	Both
1	8.00	Negative	Citrate
1	8.00	Negative	Citrate
1	8.00	Negative	Citrate
1	8.00	Negative	Citrate
1	8.00	Negative	Citrate
1	8.00	Negative	Citrate
1	8.00	Negative	Citrate

1	8.00	Negative	Citrate
1	8.00	Negative	Citrate
1	8.00	Negative	Citrate
1	8.00	Negative	Citrate
1	8.00	Negative	Both
1	8.00	Negative	Both
1	8.00	Negative	Both
1	8.00	Negative	Heparin
1	8.00	Negative	Heparin
3	9.00	Negative	Heparin
3	9.00	Negative	Heparin
3	9.00	Negative	Heparin
3	9.00	Negative	Heparin
0	9.00	Negative	Heparin
0	9.00	Negative	Heparin
0	9.00	Negative	Citrate
0	9.00	Negative	Citrate
0	9.00	Negative	Citrate
0	9.00	Negative	Citrate
0	9.00	Negative	Heparin
0	9.00	Positive	Both
0	9.00	Positive	Both
0	9.00	Positive	Both
0	9.00	Positive	Both
1	9.00	Negative	None
0	9.00	Negative	None
0	9.00	Negative	None
0	9.00	Negative	None
0	9.00	Negative	None
0	9.00	Negative	Heparin
0	9.00	Negative	Heparin
0	9.00	Negative	Heparin
0	9.00	Negative	Heparin
0	9.00	Negative	Citrate
0	9.00	Negative	Citrate
0	9.00	Negative	Citrate
0	9.00	Negative	None
0	9.00	Negative	Heparin
0	9.00	Negative	Heparin
0	9.00	Negative	Heparin
0	9.00	Negative	Heparin
0	9.00	Negative	Heparin
0	9.00	Negative	Citrate
0	9.00	Negative	Citrate
2	9.00	Negative	None
0	9.00	Positive	Citrate
0	9.00	Negative	Citrate

2	10.00	Negative	None
1	10.00	Negative	None
1	10.00	Negative	None
0	10.00	Negative	Citrate
0	10.00	Negative	Citrate
0	10.00	Negative	Citrate
0	10.00	Negative	Citrate
0	10.00	Negative	Citrate
0	10.00	Negative	Citrate
0	10.00	Negative	Citrate
0	10.00	Negative	None
0	10.00	Negative	Citrate
0	10.00	Positive	None
0	10.00	Negative	Both
0	10.00	Negative	Both
0	10.00	Negative	None
0	10.00	Negative	None
0	10.00	Negative	None
0	10.00	Negative	None
0	10.00	Negative	None
0	10.00	Negative	Both
0	10.00	Negative	Both
0	10.00	Negative	Citrate
0	10.00	Negative	Citrate
0	10.00	Negative	Citrate
0	10.00	Negative	Citrate
0	10.00	Negative	Citrate
0	10.00	Negative	None
0	10.00	Negative	None
0	10.00	Negative	Both
0	10.00	Negative	Citrate
0	10.00	Positive	Heparin
0	10.00	Positive	Both
1	11.00	Positive	Both
1	11.00	Positive	Both
0	10.00	Negative	Citrate
1	11.00	Negative	Citrate
1	11.00	Positive	Heparin
1	11.00	Negative	None
1	11.00	Negative	None
1	11.00	Negative	Citrate
0	11.00	Negative	Citrate
0	11.00	Negative	Citrate
1	11.00	Positive	Both
1	11.00	Positive	Both
1	11.00	Positive	Both
1	11.00	Positive	Both

1	11.00	Negative	Heparin
1	11.00	Negative	Heparin
1	11.00	Negative	Citrate
1	11.00	Negative	Both
1	11.00	Negative	Both
1	11.00	Negative	Both
1	11.00	Negative	Both
1	11.00	Negative	Both
1	11.00	Negative	Both
1	11.00	Negative	Both
1	11.00	Negative	Citrate
1	11.00	Negative	Citrate
1	11.00	Negative	Citrate
0	12.00	Negative	Heparin
0	12.00	Negative	Heparin
0	12.00	Negative	Citrate
0	12.00	Negative	Both
0	12.00	Negative	Both
1	12.00	Negative	Both
0	12.00	Negative	Citrate
2	12.00	Negative	Heparin
2	12.00	Negative	Heparin
0	12.00	Negative	Citrate
2	12.00	Negative	Citrate
0	12.00	Negative	Citrate
0	12.00	Negative	Heparin
0	12.00	Negative	Heparin
0	12.00	Negative	Heparin
0	12.00	Negative	Heparin
0	13.00	Negative	Citrate
0	13.00	Negative	Citrate
1	13.00	Negative	Heparin
0	13.00	Negative	Citrate
0	13.00	Negative	Citrate
0	13.00	Negative	Citrate
0	13.00	Negative	Citrate
0	13.00	Positive	Citrate
0	13.00	Positive	Citrate
0	13.00	Positive	Both
0	13.00	Positive	Both
0	13.00	Positive	Both
0	13.00	Positive	Both
0	13.00	Negative	Citrate
0	13.00	Negative	Heparin
0	13.00	Negative	Citrate
0	13.00	Negative	Citrate
0	13.00	Negative	Heparin

0	13.00	Negative	Heparin
0	13.00	Negative	Heparin
0	13.00	Negative	Heparin
0	13.00	Positive	Citrate
0	13.00	Positive	Citrate
0	13.00	Positive	Citrate
0	13.00	Positive	Citrate
0	13.00	Positive	Citrate
0	13.00	Positive	Citrate
0	13.00	Positive	Citrate
0	13.00	Negative	Citrate
1	14.00	Positive	Citrate
1	14.00	Positive	Citrate
1	14.00	Positive	Citrate
1	14.00	Positive	Citrate
1	14.00	Negative	None
1	14.00	Negative	None
1	14.00	Negative	None
1	14.00	Negative	None
1	14.00	Negative	None
1	14.00	Negative	None
1	14.00	Negative	Heparin
1	14.00	Negative	Heparin
1	14.00	Negative	Heparin
1	14.00	Negative	Citrate
1	14.00	Negative	Citrate
1	14.00	Negative	Citrate
1	14.00	Negative	Citrate
1	14.00	Negative	Citrate
1	14.00	Negative	Citrate
1	14.00	Negative	Citrate
1	14.00	Negative	Citrate
1	14.00	Negative	Citrate
1	14.00	Negative	Citrate
1	14.00	Negative	Citrate
1	14.00	Negative	Citrate
1	14.00	Negative	Both
2	14.00	Negative	Both
2	14.00	Negative	Both
2	14.00	Negative	Both
2	14.00	Negative	Both
2	14.00	Negative	Both
0	15.00	Negative	Heparin
0	15.00	Negative	Both
0	15.00	Negative	Both
1	14.00	Negative	None
0	15.00	Positive	Citrate

0	15.00	Positive	Citrate
0	15.00	Positive	Citrate
0	15.00	Negative	Citrate
0	15.00	Negative	Citrate
0	15.00	Negative	Citrate
0	15.00	Negative	Citrate
0	15.00	Negative	Citrate
0	15.00	Negative	Citrate
0	15.00	Negative	Citrate
0	15.00	Positive	Citrate
0	15.00	Positive	Citrate
0	15.00	Positive	Both
0	15.00	Positive	Both
0	15.00	Positive	Both
0	15.00	Negative	Citrate
0	15.00	Negative	Citrate
1	16.00	Negative	Heparin
1	16.00	Negative	Heparin
0	17.00	Negative	Citrate
1	18.00	Negative	Citrate
1	18.00	Negative	Citrate
0	17.00	Positive	Both
0	17.00	Positive	Both
0	17.00	Positive	Both
0	17.00	Positive	Heparin
1	18.00	Positive	Citrate
0	17.00	Positive	Citrate
1	18.00	Negative	None
1	18.00	Negative	Citrate
1	18.00	Negative	Citrate
1	18.00	Negative	Citrate
1	18.00	Negative	Citrate
0	17.00	Negative	Heparin
0	17.00	Negative	Heparin
0	17.00	Negative	Both
0	17.00	Negative	Both
2	17.00	Negative	Both
2	19.00	Negative	None
2	19.00	Negative	None
1	18.00	Positive	Both
1	18.00	Positive	Both
1	18.00	Positive	Both
1	18.00	Positive	Both
0	17.00	Negative	Citrate
0	17.00	Negative	Citrate
0	17.00	Negative	Citrate
1	18.00	Negative	Citrate

0	17.00	Negative	None
2	20.00	Positive	Citrate
2	20.00	Positive	Citrate
2	20.00	Positive	Citrate
2	20.00	Positive	Citrate
2	20.00	Positive	Citrate
0	17.00	Negative	Citrate
0	17.00	Negative	Citrate
0	17.00	Negative	Citrate
0	17.00	Negative	Citrate
0	17.00	Negative	Citrate
0	17.00	Negative	Citrate
0	17.00	Negative	Citrate
0	17.00	Negative	Citrate
0	17.00	Negative	Citrate
0	17.00	Negative	Citrate
0	17.00	Negative	Citrate
0	17.00	Negative	Citrate
0	17.00	Negative	Citrate
0	17.00	Negative	Citrate
0	17.00	Negative	Citrate
0	17.00	Negative	Both
0	17.00	Negative	Both
2	17.00	Negative	Both
2	17.00	Negative	Both
2	17.00	Negative	Both
2	17.00	Negative	Both
2	17.00	Negative	Both
2	17.00	Negative	Both
2	17.00	Negative	Both
2	17.00	Negative	Both
2	17.00	Negative	Both
2	17.00	Negative	Both
2	17.00	Negative	Both
1	18.00	Positive	Citrate
1	18.00	Positive	Citrate
1	18.00	Positive	Citrate
1	18.00	Positive	Citrate
1	18.00	Positive	Citrate
1	18.00	Positive	Citrate
1	18.00	Positive	Citrate
1	18.00	Positive	Citrate
1	18.00	Positive	Citrate
0	19.00	Negative	Citrate
0	19.00	Negative	Citrate
1	18.00	Positive	Citrate
0	17.00	Negative	Citrate

0	17.00	Negative	Citrate
0	19.00	Positive	Heparin
2	19.00	Positive	Heparin
2	19.00	Positive	Heparin
2	19.00	Positive	Heparin
2	19.00	Positive	Heparin
2	19.00	Positive	Heparin
2	19.00	Positive	Heparin
2	19.00	Positive	Heparin
2	19.00	Positive	Heparin
2	19.00	Positive	Heparin
1	18.00	Positive	Citrate
0	19.00	Positive	Citrate
0	19.00	Positive	Citrate
0	19.00	Positive	None
0	19.00	Positive	Citrate
0	19.00	Positive	Citrate
0	19.00	Positive	Citrate
0	19.00	Positive	Citrate
0	19.00	Positive	Citrate
0	19.00	Positive	Citrate
0	19.00	Positive	Citrate
0	19.00	Positive	Both
1	20.00	Negative	None
0	19.00	Negative	None
2	19.00	Negative	None
2	19.00	Negative	None
2	19.00	Negative	None
0	19.00	Negative	Both
0	19.00	Negative	Both
0	19.00	Positive	Citrate
0	19.00	Positive	Citrate
2	19.00	Positive	Citrate
2	19.00	Positive	Citrate
2	19.00	Positive	Citrate
1	19.00	Positive	Citrate
1	19.00	Positive	Citrate
2	19.00	Positive	Both
2	19.00	Positive	Both
2	19.00	Positive	Both
2	19.00	Positive	Both
2	19.00	Positive	Both
2	19.00	Positive	Both
2	19.00	Positive	Both
0	19.00	Positive	Citrate
0	19.00	Positive	Both
0	19.00	Positive	Both

2	20.00	Negative	Heparin
2	20.00	Negative	Heparin
1	18.00	Positive	Both
1	18.00	Positive	Citrate
1	18.00	Positive	Citrate
1	18.00	Positive	Citrate
1	18.00	Positive	Citrate
1	18.00	Positive	Citrate
1	18.00	Positive	Citrate
1	18.00	Positive	Citrate
1	18.00	Positive	Citrate
1	18.00	Positive	Citrate
1	18.00	Positive	Citrate
0	18.00	Positive	Citrate
0	18.00	Positive	Citrate
1	18.00	Negative	Citrate
0	19.00	Negative	Citrate
0	19.00	Negative	Citrate
